# Senescence Induces Dysfunctions in Endothelial Progenitor Cells and Osteoblasts by Interfering Translational Machinery and Bioenergetic Homeostasis

**DOI:** 10.3390/ijms19071997

**Published:** 2018-07-09

**Authors:** Guo-Shou Wang, Yung-Shuen Shen, Wen-Yi Chou, Chih-Hsin Tang, Hung-I Yeh, Li-Yu Wang, Juei-Yu Yen, Te-Yang Huang, Shih-Chia Liu, Chen-Yu Yang, Ting-Yi Lin, Chi Chen, Shih-Wei Wang

**Affiliations:** 1 Department of Orthopaedics, MacKay Memorial Hospital, Taipei 10491, Taiwan; b8905043@gmail.com (G.-S.W.); simondyh@gmail.com (T.-Y.H.); jasonscliu649@mail2000.com.tw (S.-C.L.); meinen1018@hotmail.com (C.-Y.Y.); devin100100@gmail.com (T.-Y.L.); 2 Holistic Education Center, Mackay Medical College, New Taipei City 252, Taiwan; ysshen@mmc.edu.tw; 3 Department of Orthopedic Surgery, Kaohsiung Chang Gung Memorial Hospital Medical Center, Kaohsiung 833, Taiwan; murraychou@yahoo.com.tw; 4 Department of Pharmacology, School of Medicine, China Medical University, Taichung 404, Taiwan; chtang@mail.cmu.edu.tw; 5 Chinese Medicine Research Center, China Medical University, Taichung 404, Taiwan; 6 Department of Biotechnology, College of Health Science, Asia University, Taichung 413, Taiwan; 7 Department of Medicine, Mackay Medical College, New Taipei City 252, Taiwan; yehmmc@mmc.edu.tw (H.-I.Y.); yannbo@mmc.edu.tw (L.-Y.W.); u948-009@mmc.edu.tw (J.-Y.Y.); 8 Department of Internal Medicine, MacKay Memorial Hospital, Taipei 10491, Taiwan; 9 Department of Education and Research, Taipei City Hospital Renai Branch, Taipei 106, Taiwan; 10 Graduate Institute of Natural Products, College of Pharmacy, Kaohsiung Medical University, Kaohsiung 807, Taiwan

**Keywords:** endothelial progenitor cell, osteoblast, senescence, Akt/mTOR/p70S6K, ATP synthesis

## Abstract

Age-related bone diseases are partly caused by impaired bone integrity, which are closely related to osteoblasts’ activity and angiogenesis. Endothelial progenitor cells (EPCs) are the initiators of angiogenesis and found to have senescent-induced dysfunctions. The aim of this study is to investigate the effects of senescence in EPCs on osteogenesis and angiogenesis. Human primary EPCs and a murine osteoblast cell line (MC3T3-E1) are utilized in this study. The senescence of EPCs are induced by serial passages. When co-cultured with senescent EPCs, the osteoblasts demonstrate weakened alkaline phosphatase (ALP) activity and mineral deposition. On the other hand, osteoblast-induced migration decreases in senescent EPCs. As for the intracellular alterations of senescent EPCs, the activation of Akt/mTOR/p70S6K pathway, MnSOD and catalase are diminished. In contrast, the level of reactive oxygen species are significantly higher in senescent EPCs. Furthermore, senescent EPCs has decreased level intracellular ATP level and coupling efficiency for oxidative phosphorylation while the non-mitochondrial respiration and glycolysis are elevated. The senescence of EPCs impairs the functions of both osteoblasts and EPCs, suggesting EPCs’ role in the pathophysiology of age-related bone diseases. Targeting the alterations found in this study could be potential treatments.

## 1. Introduction

Self-maintenance and regeneration are essential for bone tissue to sustain its function, and may be weakened in the elderly [1]. The integrity of bone is maintained through consecutive dynamic balance between bone resorption and mineralization, which are separately mediated by osteoclasts and osteoblasts [2]. Such equilibrium is disturbed in the elderly, resulting changes in bone’s structure and its ability to regenerate [1,3,4,5]. These alterations could result in osteoporosis, fracture and nonunion, leading to frailty, morbidity and mortality in older population [6,7,8,9].

Impaired bone regeneration play a critical role in osteoporosis and nonunion [10]. The detailed mechanism of this phenomenon is not fully understood but it is believed to be closely related to osteoblasts and their interactions with other types of cells and tissues. For example, osteoblasts need to interact with vascular tissue since the vasculature not only serves as the nutrient-metabolite exchanging passage for bone formation, but also supports the self-renewing osteoprogenitors with suitable niches [11,12]. Recent studies focusing on osteoporotic disease and healing process of fractures have shown the importance of the osteogenic-angiogenic interface [13]. Angiogenesis is a process of new blood vessel forming from existing vessels. The initiation of angiogenesis is mediated by circulating endothelial progenitor cells (EPCs) deriving from bone marrow [14]. EPCs are cells with the ability to launch angiogenesis in several physiological or pathological situations, such as embryonic growth, cardiovascular dysfunction, tumor formation and fracture healing [15,16,17,18]. Previous studies have shown increased amount of circulating EPCs in peripheral blood of patients with bone fracture compared with healthy control subjects [19,20,21]. Furthermore, EPCs implantation has been found to boost fracture healing in animal models [22]. One of the possible mechanisms that involves in the regeneration of bone is the collaboration of EPCs and osteoblasts. There are increasing studies suggesting that there are reciprocal angiogenic/osteogenic interactions between these two types of cells. In hypoxic conditions, osteoblasts can secrete proangiogenic factors, including vascular endothelial growth factor (VEGF), which can also be secreted by EPCs [23,24]. The maturation of both osteoblasts and EPCs can be significantly facilitated by these factors, suggesting that osteoblasts and EPCs may promote proliferation within each other, and encourage activity through the intercellular signaling [25,26]. However, senescent EPCs have been found to have impairments in multiple physiological activities, such as the migratory and differentiation capacities [27,28]. Whether the interaction between osteoblasts and EPCs is related to the increased bone diseases in older population remains largely unknown.

This study utilized the co-culture model, in which osteoblasts and young or senescent EPCs were cultured together, to better understand the impact of senescence on EPCs regarding their interaction with osteoblasts. The evaluations of several cellular functions of osteoblasts and EPCs were conducted. For osteoblasts, the activity of alkaline phosphatase (ALP) and the level of calcium deposition were measured after co-cultured with young or senescent EPCs. For EPCs, effect of senescence on EPCs’ migration when co-cultured with osteoblast was evaluated. This study also examined the intracellular expression level of Akt/mTOR/p70S6K signaling pathway, which governs the angiogenic activities of EPCs via regulating protein translation [29]. An additional focus of this study was to analyze alterations of bioenergetics of senescent EPCs by measuring the oxygen consumption rates and other related measurements of young and senescent EPCs.

## 2. Results

### 2.1. EPCs with Serial Passaging Express Characters of Senescence

**Figure 1:**
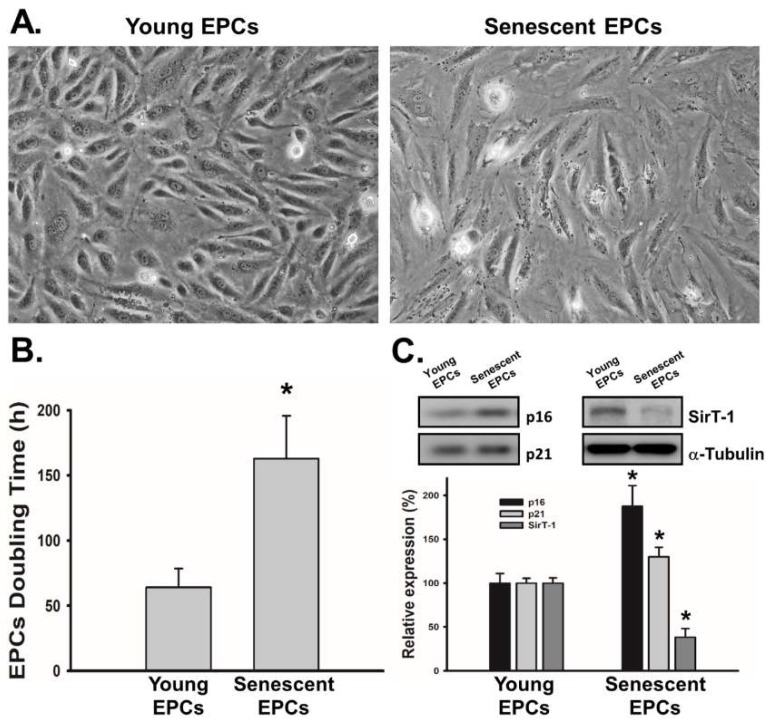
Senescence of endothelial progenitor cells (EPCs) was induced by serial passages. To obtain young and senescent EPCs, cells were grown in culture medium and serially passaged until their passage difference results in doubled cell doubling time. (**A**) The representative morphology of young and senescent EPCs was shown (phase contrast, 100×); (**B**) Cell doubling time was increased at least 2-fold in senescent EPCs after serial passages (*N* = 6); (**C**) For characterization of senescence in EPCs, the expression of senescence marker p16, p21 and sirtuin 1 (SirT-1) was determined by Western blot analysis (*N* = 6). Data are expressed as mean ± S.E.M. of six independent experiments. * *p* < 0.05 compared with the group of young EPCs.

To validate the liability of the serial passage model for senescence, we examined the indicators of EPCs’ senescence, including morphology, doubling time and molecular markers for senescence. After serial passages of EPCs culture, we observed morphological changes and significantly prolonged doubling time in EPCs with higher passage number (Figure 1A,B). Senescent EPCs were less cobblestoned-shaped than young EPCs while their doubling time was nearly 2.5-fold than young ones. Next, we examined expression levels of senescence markers, including p16, p21 and sirtuin 1 (SirT-1). Results demonstrated that, compared to EPCs with lower passage number, EPCs with higher passage number had higher expression level of p16 and p21, while the expression of SirT-1 level was lower (Figure 1C). This profile of senescence markers was identical with previous studies [27,30,31,32].

### 2.2. EPCs’ Senescence Represses Bone Formation of Osteoblasts

**Figure 2:**
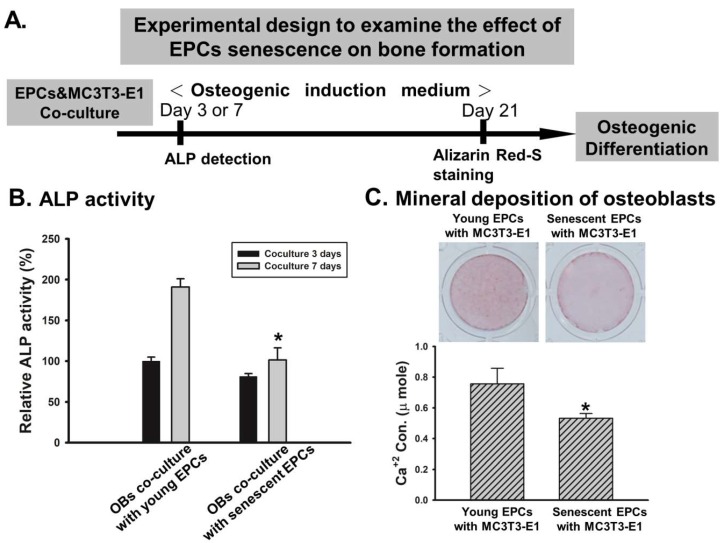
Effect of EPCs senescence on osteogenic function of osteoblasts. (**A**) Schematic diagram of the experimental design for EPCs and osteoblasts co-culture model. Murine osteoblast cell line (MC3T3-E1) cells were grown in co-culture with young or senescent EPCs, then incubated in the osteogenic induction medium for bone formation for the indicated times; (**B**) Alkaline phosphatase (ALP) activity of MC3T3-E1 cells decreased in co-culture with senescent EPC on day 3 and day 7 (*N* = 5); (**C**) Calcium deposition was decreased in MC3T3-E1 cells after co-culture with senescent EPC for 21 days (*N* = 5). Data are expressed as mean ± S.E.M. of five independent experiments. * *p* < 0.05 compared with the group of young EPCs.

We evaluated the effect of EPCs on bone-forming ability of a murine osteoblast cell line (MC3T3-E1) by EPCs/osteoblasts co-culture model (Figure 2A). We found that both ALP activity and calcium deposition of MC3T3-E1 decreased when cultured with senescent EPCs (Figure 2B,C). The ALP activity of MC3T3-E1 cultured with young EPCs, almost doubled by day 7 of co-culture, compared with the ALP activity at day 3. In contrast, the ALP activities of MC3T3-E1 cultured with senescent EPCs were significantly reduced at both day 3 and day 7 of co-culture. Similar trends could be detected at the Alizarin Red-S staining, which show minimal mineral deposition of MC3T3-E1 when cultured with senescent EPCs.

### 2.3. Senescence Impairs Osteoblast-Attracted EPCs Migration

**Figure 3:**
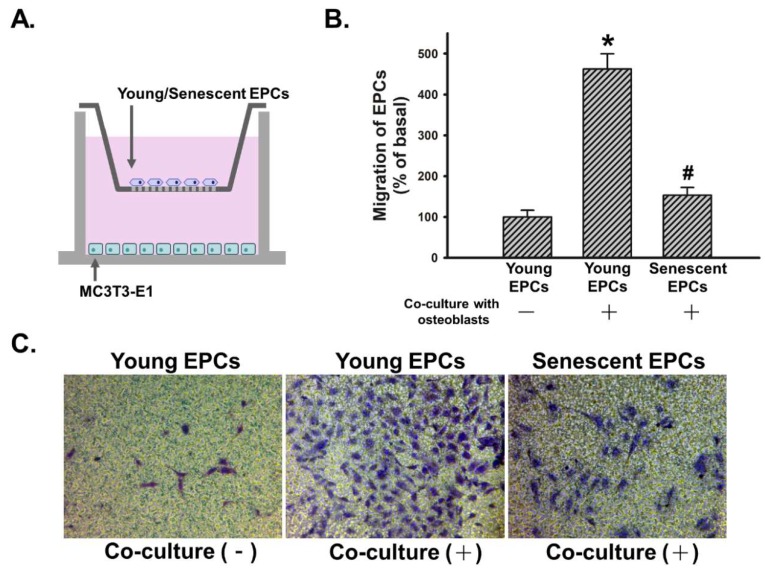
Effect of senescence on osteoblast-attracted EPCs migration. Young and senescent EPCs were seeded onto an upper chamber, then co-culture with or without MC3T3-E1 cells and migration activity of EPCs was measured after 24 h. (**A**) Scheme of transwell co-culture model for EPCs and MC3T3-E1 cells; (**B**) Cells that migrated the filter were counted and quantified (*N* = 5) as mean ± S.E.M. * *p* < 0.05 compared with the basal group (without co-culture). # *p* < 0.05 compared with the group of young EPCs; (**C**) Representative images of migrated EPCs were shown (phase contrast, 40×).

We evaluated the effect of osteoblast on migratory activity of EPCs, which is an indicator for EPCs’ initiation of angiogenesis, by co-culturing MC3T3-E1 with young or senescent EPCs in a transwell migration model (Figure 3A). In the absence of MC3T3-E1, EPCs did not actively migrate through the permeable membrane between two chambers. Meanwhile, young EPCs’ migration was stimulated while senescent EPCs demonstrated weakened migration in the co-culture model. Osteoblast-induced migratory activity of young EPCs was over two times higher than that of senescent EPCs (Figure 3B,C).

### 2.4. Senescence Inhibits OBCM-Induced Akt/mTOR Translational Pathway in EPCs

**Figure 4:**
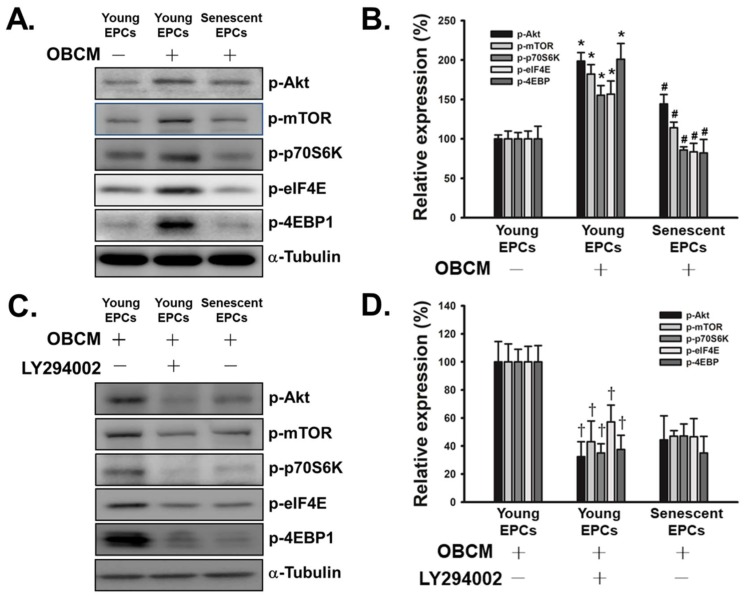
Effect of senescence on osteoblast conditioned medium (OBCM) activated Akt/mTOR translational pathway in EPCs. (**A**,**B**) Young and senescent EPCs were treated with or without OBCM of MC3T3-E1 cells for 24 h (*N* = 5); (**C**,**D**) Young and senescent EPCs were treated with OBCM in the absence or presence of LY294002 (10 µM) for 24 h (*N* = 5). After treatment, cells were harvested and lysed for the detection of p-Akt, p-mTOR, p-p70S6K, p-eIF4E, p-4E-BP1, and *α*-tubulin by Western blot analysis. The quantitative densitometry of the relative level of signals was performed with Image-Pro Plus. Data are expressed as mean ± S.E.M. of five independent experiments. * *p* < 0.05 compared with the basal group (without OBCM). # and † *p* < 0.05 compared with the group of young EPCs.

We then investigated the potential signaling pathway related to EPCs’ effect on osteoblasts and their own migratory activity (Figure 4). Previous studies have shown that Akt/mTOR/p70S6K pathway is the downstream of VEGF and related to mobilization of EPCs [33,34,35]. As shown in Figure 4A,B, osteoblast conditioned medium (OBCM) activated Akt/mTOR/p70S6K pathway in young EPCs, with the level of phosphorylated Akt, mTOR, p70S6K, eukaryotic translation initiation factor 4E (eIF4E) and eukaryotic translation initiation factor 4E-binding protein 1 (4E-BP1) significantly elevated. However, such activation did not appear among senescent EPCs when treated with OBCM. Furthermore, by administering phosphoinositide 3-kinase (PI3K) inhibitor, LY294002, the activation of Akt/mTOR/p70S6K pathway in young EPCs was inhibited, indicating OBCM-induced activation of Akt/mTOR/p70S6K pathway was mediated by PI3K (Figure 4C,D).

### 2.5. Senescence Alters OBCM-Mediated Reduction-Oxidation Balance in EPCs

**Figure 5:**
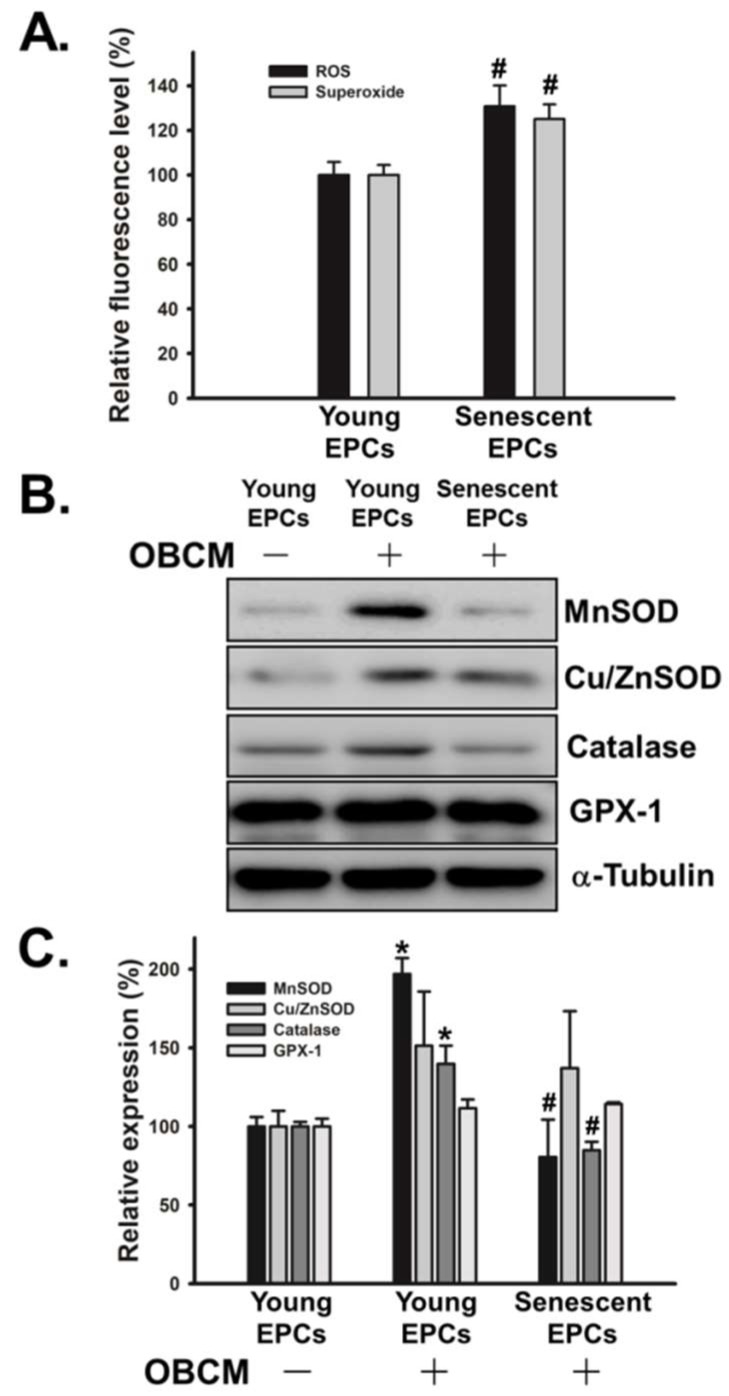
Effect of senescence on the content of ROS, superoxide, and antioxidant enzymes in OBCM-treated EPCs. (**A**) Young and senescent EPCs were incubated with OBCM of MC3T3-E1 cells for 24 h. Then, the production of ROS and superoxide was examined by ROS/superoxide detection kit (*N* = 5); (**B**,**C**) Young and senescent EPCs were treated with or without OBCM of MC3T3-E1 cells for 24 h. After treatment, cells were harvested and lysed for the detection of antioxidant enzymes by Western blot analysis (*N* = 5). The quantitative densitometry of the relative level of signals was performed with Image-Pro Plus. Data are expressed as mean ± S.E.M. of five independent experiments. * *p* < 0.05 compared with the basal group (without OBCM). # *p* < 0.05 compared with the group of young EPCs.

Another frequently discussed issue of senescence is the imbalance of intracellular reduction-oxidation balance. First, we found that senescence significantly increased the production of reactive oxygen species (ROS) and superoxide in OBCM-treated EPCs (Figure 5A). In addition, we analyzed the reduction-oxidation status by examining the level of antioxidant enzymes in OBCM-stimulated EPCs. The results showed that senescence did not affect the level of Cu/ZnSOD and GPX-1, but did dramatically suppress the expression of MnSOD and catalase (Figure 5B,C).

### 2.6. Senescence Disrupts OBCM-Regulated Bioenergetic Homeostasis in EPCs

**Figure 6:**
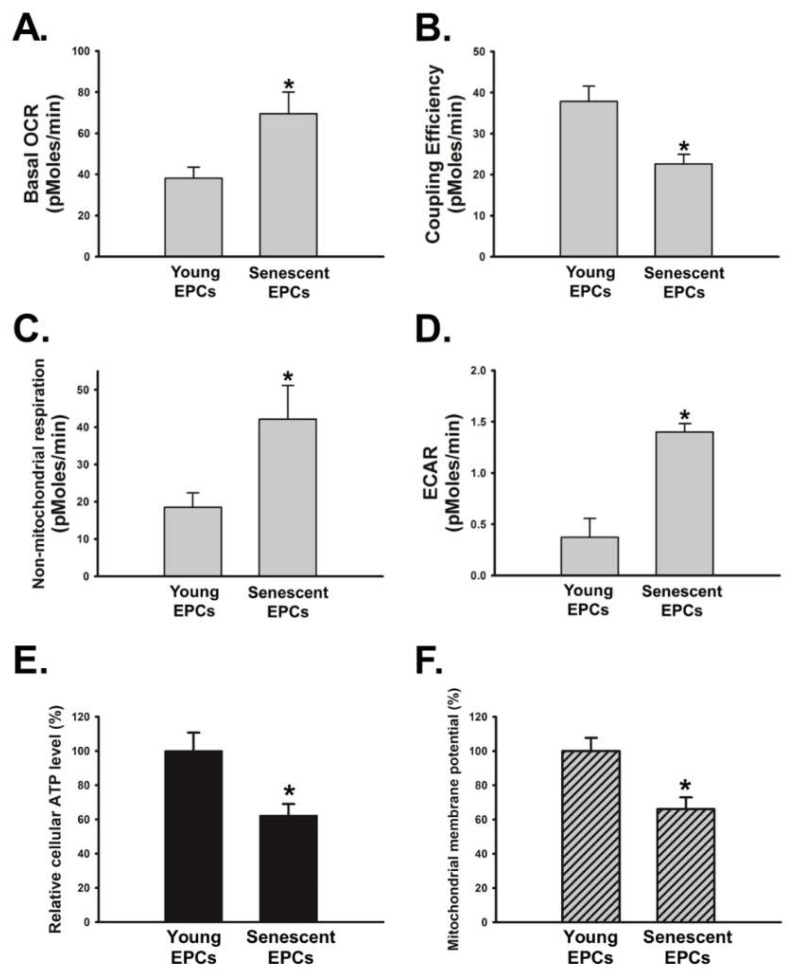
Effect of senescence on bioenergetic homeostasis in OBCM-treated EPCs. Young and senescent EPCs were incubated with OBCM of MC3T3-E1 cells for 24 h. (**A**–**D**) Oxygen consumption rate (OCR) and extracellular acidification rate (ECAR) were determined by the Seahorse XF24 analyzer. Meanwhile, coupling efficiency and non-mitochondria respiration were assessed by bioenergetic analysis in cells exposed to mitochondrial component inhibitors using XF24 analyzer (*N* = 5); (**E**,**F**) The ATP synthesis and mitochondrial membrane potential of EPCs were examined by ATP detection assay and Mito-ID^®^ assay kit, respectively (*N* = 5). Data are expressed as mean ± S.E.M. of five independent experiments. * *p* < 0.05 compared with the group of young EPCs.

Alterations of both Akt/mTOR/p70S6K pathway and intracellular reduction-oxidation balance could lead to changes in cellular metabolism [36,37]. Therefore, we examined the dynamic change of oxygen consumption rate (OCR) and extracellular acidification rate (ECAR) in OBCM-treated EPCs. We found that basal OCR was higher in the senescent EPCs (Figure 6A). However, young EPCs had higher coupling efficiency than senescent EPCs (Figure 6B), which is an index of proton conductance and mitochondrial dysfunction [38]. Non-mitochondria respiration and extracellular acidification rate (ECAR), which is an indicator of activity of non-mitochondrial NADPH oxidases and glycolysis [38], respectively, were higher in senescent EPCs when compared with young EPCs (Figure 6C,D). Further investigation of cellular energetics showed significantly decreased cellular ATP and mitochondrial membrane potential in senescent EPCs when incubated with OBCM (Figure 6E,F).

## 3. Discussion

Senescent EPCs have compromised interactions with osteoblasts. To our knowledge, this is the first report of such a phenomenon. This study revealed that the bone formation, including ALP activation and calcium deposition, of osteoblasts was boosted by young EPCs but not senescent EPCs. Furthermore, EPCs’ reactions upon osteoblasts’ stimulation, including migration, intracellular Akt/mTOR/p70S6K protein translation-regulating pathway, expression of antioxidants and cellular respiration, were significantly altered in senescent cells.

Previous studies have shown aging effect caused by prolonged culture of EPCs [39]. To evaluate the aging effect of EPCs, EPCs from same donor but with different passage numbers were used. This study examined the cellular aging profile, including SirT-1, p16 and p21, of EPCs with higher passage number, and found that the profile is consistent with that of elderly EPCs reported in previous studies (Figure 1C) [27,30,31,32]. SirT-1 is a nicotinamide adenine dinucleotide-dependent deacetylase with anti-aging effects. Furthermore, it can assist initiating angiogenesis by activating endothelial nitric oxide synthase (eNOS) [40]. p16 and p21 are cyclin-dependent kinase inhibitors, which prevent cells from replication and arrest the cell cycle in G1 through interacting with p53 in EPCs [40,41]. Our data showed decreased expression of SirT-1 and increased expressions of p16 and p21, validating our model for the EPCs’ aging effect on osteogenesis.

One of the most critical steps in the mineralization of osteogenesis is the deposition of hydroxyapatite in the extracellular matrix. This process can be inhibited by inorganic pyrophosphate, which can be hydrolyzed by membrane-bound ALP expressed by osteoblasts. In addition, inorganic phosphate derived from hydrolysis of inorganic pyrophosphate can combine with calcium to form hydroxyapatite [42]. This study firstly demonstrated strengthened osteoblast/EPCs-induced osteogenesis, and found that the ALP activity and calcium deposition of MC3T3-E1 were significantly decreased when cultured with senescent EPCs (Figure 2B,C). Furthermore, senescent EPCs shows less impact on osteoblasts compared to young EPCs. A recent study with similar results showed that when culturing bone marrow mesenchymal stromal cells and peripheral blood mononuclear cells, which are the source of osteoprogenitors and EPCs, respectively, increased ALP expression and calcium concentration can be detected [43]. In addition, this study also showed that these reactions diminished among the cells from older donors.

EPCs can mediate angiogenesis and vascular repair through proliferations, and differentiation into endothelial cells after being released from their niches and locating to the target regions. Such recruitment can be caused by vascular injury or physiological stress, such as a bone fracture [20,44]. Quiescent EPCs can be activated by multiple growth factors and chemokines, including VEGF and SDF-1. Both of these molecules are secreted by osteoblasts during bone formation [17,24,44,45]. In this study, the ability of osteoblasts to recruit EPCs through paracrine signaling was confirmed in the transwell migration assay (Figure 3C). Moreover, senescent EPCs had an attenuated reaction in this co-culture model (Figure 3B).

To investigate the mechanism of reduced migration in senescent EPCs, the phosphorylations of Akt/mTOR/p70S6K/4E-BP1 signaling pathway, which is the downstream of VEGF and SDF-1, were measured [33,35]. Activation of this translation-mediating signaling pathway can promote the mobilization of EPCs to peripheral circulation partly by upregulating MMP-9 [34,44]. When cultured with osteoblast conditioned medium, the Akt/mTOR/4E-BP1 pathway in quiescent young EPCs was activated while there was almost no response in senescent EPCs (Figure 4A,B). Such impaired signal transduction could explain both the prolonged doubling time and attenuated migration of senescent EPCs, as Akt/mTOR pathway affects cell migration and cell proliferation [46].

ROS are compounds produced during the cellular metabolism and believed to be one of the causes of the senescent phenotype in endothelial cells. Recent studies have shown that, besides causing damage to DNA, proteins, and lipids through oxidation, ROS are important regulators in the process of angiogenesis. ROS effect several critical cell signaling pathway, including PI3K/Akt, p16, GPCR, Notch, JAK-STAT and MAPK [40,47]. In our study, we demonstrated that the level of intracellular ROS was around 1.2 times higher in senescent EPCs than those in young EPCs (Figure 5A). We also found low expression levels of multiple antioxidant enzymes in senescent EPCs. This might be one of the causes of elevated ROS level (Figure 5B,C). 

The abnormal ROS level in senescent cells can cause mitochondrial dysfunction and lead to impaired electron transport chain, especially complex I and IV [36,37]. This study found that senescent EPCs had decreased intracellular ATP (Figure 6E). Despite having high oxygen consumption rate, senescent EPCs had lower coupling efficiency and mitochondrial membrane potential compared to young EPCs (Figure 6A,B,F). Previous studies have suggested that senescent cells with ATP insufficiency may increase their cellular oxygen consumption as compensation [37]. In addition, the senescent EPCs had increased non-mitochondrial respiration and glycolysis, which might possibly be the compensatory reactions for the ATP insufficiency (Figure 6C,D).

Dysfunctions of antioxidants and electron transport chain are the characteristics of an aging cardiovascular system [48,49]. These defects not only disrupt the bioenergetics of the cell, but also have interactions with Akt/mTOR signaling pathway. Previous studies have found that elevated ROS can activate Akt signaling pathway in endothelial cells; however this phenomenon was not observed in the EPCs of this study [37]. One of the possible explanations would be that the mechanism allowing ROS to activate Akt was also impaired in senescent EPCs. On the other hand, mTOR have been found to have the ability to enhance mitochondrial membrane potential and ATP synthetic capacity, through forming complexes with Bcl-xl and VDAC1 directly on the outer-membrane of mitochondria [50,51]. In our study, senescent EPCs failed to activate mTOR upon the stimulus of osteoblast and had mitochondrial dysfunction, such as reduced intracellular ATP, impaired coupling efficiency and decreased membrane potential. This is consistent with previous studies. Furthermore, the inhibition of mTOR can also augment aerobic glycolysis, which may partly explain the elevated non-mitochondrial respiration and glycolysis in senescent EPCs [51].

Recent studies found that in certain pathological conditions, such as metastatic prostate cancer and fibrodysplasia ossificans progressiva, endothelial cells can be converted to osteoblasts [52,53]. Such findings indicate a tight relationship between the osteoblast and endothelial cell lineage. However, similar interactions still require extensive investigation in physiological conditions. In the present study, osteoblasts demonstrated significant ability to recruit EPCs (Figure 3). Whether the recruitment could be followed by further differentiation, such as EPCs to endothelial cells or even to osteoblasts, remains unknown and warrants further in vitro and in vivo studies.

There were several limitations in this study. First, cultured MC3T3-E1, which is a murine osteoblast cell line, was utilized with human EPCs in the experiments. Although MC3T3-E1 is not exactly the same as human primary osteoblasts, previous studies have validated the consistency between MC3T3-E1’s and the primary osteoblasts in terms of the characteristics of bone formation and shown that MC3T3-E1 can interact with human mesenchymal stem cells and growth factors [54,55,56]. Another limitation is that we did not clarify the detailed components secreted by both EPCs and osteoblasts. VEGF may play an important role in this process; however, a previous study has demonstrated that VEGF alone cannot explain the enhanced bone formation when culturing osteogenic progenitors and angiogenic progenitors [43]. Therefore, it is possible that there could be complex elements in their interactions, which require further investigation.

**Figure 7:**
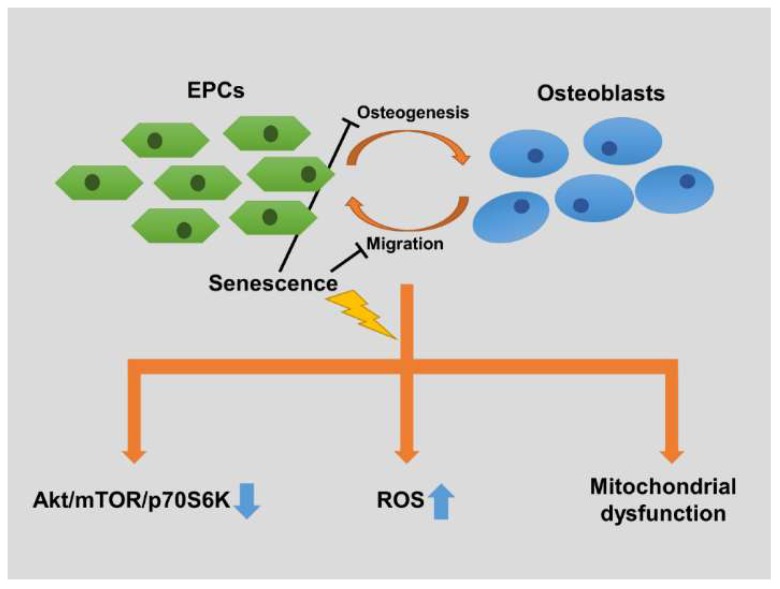
Schematic presentation of mechanism underling senescence-induced dysfunctions in endothelial progenitor cells and osteoblasts. Osteoblasts osteogenic activity and EPCs migratory activity both decreased in the co-culture system using senescent EPCs. Senescent EPCs decreased activation of Akt/mTOR/p70S6K signaling pathway, and elevated intracellular reactive oxygen species (ROS) and mitochondrial dysfunction (including decreased intracellular ATP and mitochondrial coupling efficiency) when co-cultured with osteoblasts.

In conclusion, this study demonstrated three key points (Figure 7): (1) senescent EPCs have less ability to stimulate osteoblasts and reduced reaction to osteoblasts. Both osteogenesis and EPCs’ migration are less robust when the EPCs are senescent; Secondly, (2) the osteoblast-induced activation of Akt/mTOR/4E-BP1 pathway in senescent EPCs is diminished; Finally, (3) there are significant senescence-induced alterations in EPCs’ bioenergetics, including redox imbalance and inefficient mitochondrial respiration. These findings could provide new insights into age-related bone diseases, and help develop effective treatments and prophylaxes for the elderly.

## 4. Materials and Methods

### 4.1. Cell Culture

Ethical approval was granted by the Institutional Review Board of Mackay Medical College, New Taipei City, Taiwan (reference number: No. P990001). Informed consent was obtained from healthy donors before the collection of peripheral blood (80 mL). Demographic information of EPCs donors is shown in Table S1. The peripheral blood mononuclear cells (PBMCs) were fractionated from other blood components by centrifugation on Ficoll-Paque plus (Amersham Biosciences, Uppala, Sweden) according to the manufacturer’s instructions. CD34-positive progenitor cells were obtained from the isolated PBMCs using CD34 MicroBead kit and MACS Cell Separation System (Miltenyi Biotec, Bergisch Gladbach, Germany). The maintenance and characterization of CD34-positive EPCs were performed as described previously [57]. Briefly, human CD34-positive EPCs were cultured and propagated in MV2 complete medium consisting of MV2 basal medium and growth supplement (PromoCell, Heidelberg, Germany), supplied 20% defined FBS (HyClone, Logan, UT, USA). The cultures were seeded onto 1% gelatin-coated plasticware and maintained at 37 °C in a humidified atmosphere of 5% CO_2_. The murine osteoblast cell line MC3T3-E1 was purchased from American Type Culture Collection (ATCC, Manassas, VA, USA). MC3T3-E1 cells were cultured in α-MEM supplemented with 10% FBS and antibiotics (100 U/mL penicillin and 100 µg/mL streptomycin) at 37 °C with 5% CO_2_. The passage numbers of MC3T3-E1 used in this study were between P10 to P15.

### 4.2. Obtainment of Senescent EPCs

To establish in vitro aging model of human EPCs, EPCs were grown in culture medium and underwent serial passages. Senescent EPCs were defined as an increase of cell doubling time for more than 100% of the control cells, which were less than passage 8. Measurement of cell doubling time was determined by staining trypan blue dye (Sigma-Aldrich, St. Louis, MO, USA), and counted in duplicate using hemocytometer. The passage difference between young and senescent EPCs used in this study was at least 7 passages.

### 4.3. Co-Culture Model

For the direct cell contact model, MC3T3-E1 osteoblast cells (3 × 10^4^ cells/well) were grown in co-culture with young or senescence EPCs (3 × 10^4^ cells/well) in MV2 complete medium supplemented with 10% FBS for various time periods using 24-well plates. After an overnight co-culture, the medium was changed to osteogenic induction medium (α-MEM containing vitamin C 50 μg/mL and β-glycerophosphate 10 mM), then the osteogenic function of osteoblasts was evaluated by ALP activity assay and mineral deposition analysis.

### 4.4. .Measurement of ALP Activity

After co-culture for 3 and 7 days, incubated cells, including MC3T3-E1 cells, were harvested or corrected in 0.2% Nonidet P-40 and the cell suspension was disrupted by sonication. After centrifugation, ALP activity in the supernatant was measured by ALP activity kit (Sigma-Aldrich, St. Louis, MO, USA).

### 4.5. Mineral Deposition Analysis

MC3T3-E1 and EPCs were co-cultured in osteogenic induction medium for 3 weeks and the medium was changed every 3 days. After incubation for 21 days, cells were washed twice with 20 mM Tris buffered saline (TBS) containing 0.15 M NaCl (pH 7.4) and fixed in ice-cold 75% ethanol for 30 min, and then air-dried. Calcium deposition was determined using alizarin red-S staining. The ethanol-fixed cells and matrix were stained for 1 h with 40 mM alizarin red-S. (pH 4.2) and extensively rinsed with water.

### 4.6. EPCs Migration Assay

The migration assay was performed using Transwell 24-well dishes with a pore size of 8 μm (Costar, New York, NY, USA). EPCs (3 × 10^4^ cells/well) were seeded onto the upper chamber with MV2 basal medium, then the inserts were placed in these wells containing MC3T3-E1 (2 × 10^5^ cells) with serum-free medium for co-culture to attract EPCs migration. After 24 h of treatment, EPCs on the upper side of the filters were mechanically removed. Those that migrated on the lower side were fixed with 4% formaldehyde, then stained with 0.5% crystal violet for 10 min. Finally, migration cells were quantified by counting the number of stained cells in 10 random fields with the inverted phase contrast microscope and photographed.

### 4.7. Preparation of Osteoblast Conditioned Medium (OBCM)

Murine osteoblast cells (MC3T3-E1) were cultured to 90% confluence in 10 cm dish. Cells were then washed and changed to serum-free medium. OBCM was subsequently collected 2 days after the change of medium and stored at −80 °C for all experiments.

### 4.8. Determination of Reactive Oxygen Species (ROS) and Superoxide Production

EPCs were seeded in black 96-well plates at a density of 2 × 10^4^ cells per well. After 24 h of incubation, the culture medium was removed and treated with OBCM for 24 h. ROS and superoxide production were then determined in live cells using ROS/superoxide detection kit (Enzo Life Sciences, Plymouth Meeting, Montgomery, PA, USA) according to the manufacturer’s instruction. The levels of ROS were monitored at Ex/Em: 488/520 nm, meanwhile superoxide production was detected at Ex/Em: 550/610 nm using FlexStation 3 microplate reader (Molecular Devices, Sunnyvale, CA, USA).

### 4.9. Western Blot Analysis

The cellular lysates were prepared according to our previous instruction [58]. Proteins were resolved on SDS-PAGE and transferred to PVDF membranes. The blots were blocked with 4% BSA for 1 h at room temperature and then probed with indicated primary antibodies for 1 h at room temperature. Then, the blots were subsequently incubated with the secondary antibody for 1 h at room temperature. Finally, the signals were determined using the chemiluminescent assay kit (Amersham Biosciences, Buckinghamshire, UK). 

### 4.10. Bioenergetic Function Analysis

The XF24 Analyzer (Seahorse Bioscience, North Billerica, MA, USA) was used to determine the bioenergetic function of EPCs. After treatment, EPCs (2 × 10^4^ cells/well) were incubated on ice for 10 min with 330 µL of assay buffer (125 mM sucrose, 65 mM KCl, 2 mM MgCl_2_, 20 mM phosphate buffer, pH 7.2) in 24-well plates. The plate was then transferred into the incubation chamber, and the oxygen consumption rate (OCR) and extracellular acidification rate (ECAR) were monitored real-time by XF24 Analyzer at 37 °C. The program of Seahorse XF24 Analyzer was set according to the manufacturer’s recommendation and the data are expressed in pmol/min/10^4^ cells for OCR and in mpH/min/10^4^ cells for ECAR to allow comparison between independent experiments.

### 4.11. Measurement of Intracellular ATP Content

Intracellular ATP level was measured by luminescent ATP detection assay (Abcam, Cambridge, UK). After treatment of OBCM, 50 µL of detergent was added to 100 µL of media in each well to release the intracellular ATP. Half of the mixture was then transferred into a black 96-well plate containing 50 µL of substrate solution, and the luminescence intensity was measured using FlexStation 3 (Molecular Devices, Sunnyvale, CA, USA).

### 4.12. Mitochondrial Membrane Potential (MMP) Analysis

The changes of MMP was analyzed by Mito-ID^®^ assay kit (Enzo Life Sciences, Plymouth Meeting, Montgomery, PA, USA). EPCs (2 × 10^4^ cells/well) were seeded onto black 96-well plates. Overnight, the culture medium was removed and cells were incubated with OBCM for 24 h. After treatment, Mito-ID^®^ MP detection reagent was added directly into the medium for 30 min at 37 °C. Then, MMP was observed immediately at Ex = 490 nm/Em = 590 nm using fluorescence microplate reader (FlexStation 3).

### 4.13. Statistical Analyses 

Data are presented as the mean ± S.E.M. for the indicated number of separate experiments. Statistical analyses of data were done with one-way analysis of variance followed by Student’s *t*-test. The difference is significant if the *p*-value is <0.05.

## Supplementary Materials

Supplementary materials can be found at http://www.mdpi.com/1422-0067/19/7/1997/s1.
