# Supplementary material for: Senescence Induces Dysfunctions in Endothelial Progenitor Cells and Osteoblasts by Interfering Translational Machinery and Bioenergetic Homeostasis
Source: Int J Mol Sci. 2018 Jul 9;19(7):1997. doi: 10.3390/ijms19071997 (PMC6073720; doi:10.3390/ijms19071997)
Supplement: Supplementary file 1 [file ijms-19-01997-s001.pdf]

Table S1. Demographic information of endothelial progenitor cells (EPCs) donors.

No. of EPC Clones	Gender #	Age (Year)	Experiment *
**11B39**	M	20	Exp1
**11D30**	M	20	Exp1, Exp2, Exp3, Exp4, Exp5, Exp6
**11D46**	M	20	Exp2
**11D67**	M	25	Exp3, Exp4, Exp5, Exp6
**11D75**	M	29	Exp1, Exp2, Exp3, Exp4, Exp5, Exp6
**11E48**	M	25	Exp2

M = Male. * Each experiment was replicated with at least 3 different clones of EPCs. Experiment 1 (Exp1): Aging of EPCs, shown in Figure 1. Experiment 2 (Exp2): Co-culture of EPCs and murine osteoblast cell line (MC3T3-E1), shown in Figure 2. Experiment 3 (Exp3): Transwell chamber assay, shown in Figure 3. Experiment 4 (Exp4): Cellular signal transduction analysis, shown in Figure 4. Experiment 5 (Exp5): Cellular redox analysis, shown in Figure 5. Experiment 6 (Exp6): Bioenergetics analysis of EPCs, shown in Figure 6.
